# Advance care planning and outcome in pediatric palliative home care

**DOI:** 10.18632/oncotarget.24929

**Published:** 2018-04-03

**Authors:** Jessica I. Hoell, Hannah L. Weber, Stefan Balzer, Mareike Danneberg, Gabriele Gagnon, Laura Trocan, Arndt Borkhardt, Gisela Janßen, Michaela Kuhlen

**Affiliations:** ^1^ University of Duesseldorf, Medical Faculty, Department of Pediatric Oncology, Hematology and Clinical Immunology, Center for Child and Adolescent Health, Duesseldorf, Germany

**Keywords:** terminal care, advance care planning (ACP), pediatric palliative care, palliative medicine, medical orders for life-sustaining treatment (MOLST)

## Abstract

Pediatric advance care planning seeks to ensure end-of-life care conforming to the patients/their families’ preferences. To expand our knowledge of advance care planning and “medical orders for life-sustaining treatment” (MOLST) in pediatric palliative home care, we determined the number of patients with MOLST, compared MOLST between the four “Together for Short Lives” (TfSL) groups and analyzed, whether there was a relationship between the content of the MOLST and the patients’ places of death.

The study was conducted as a single-center retrospective analysis of all patients of a large specialized pediatric palliative home care team (01/2013-09/2016). MOLST were available in 179/198 children (90.4%). Most parents decided fast on MOLST, 99 (55.3%) at initiation of pediatric palliative home care, 150 (83.4%) within the first 100 days. MOLST were only changed in 7.8%. Eighty/179 (44.7%) patients decided on a Do Not Attempt Cardio-Pulmonary Resuscitation (DNACPR) order, 58 (32.4%) on treatment limitations of some kind and 41 (22.9%) wished for the entire spectrum of life-sustaining measures (Full Code). Most TfSL group 1 families wanted DNACPR and most TfSL group 3/4 parents Full Code. The majority (84.9%) of all DNACPR patients died at home/hospice. Conversely, all Full Code patients died in hospital (80% in an intensive care setting).

The circumstances of the childrens’ deaths can therefore be predicted considering the content of the MOLST. Regular advance care planning discussions are thus a very important aspect of pediatric palliative home care.

## INTRODUCTION

The main goal of pediatric palliative home care (PPHC) in children and their families with life-limiting conditions (LLCs) is the best possible quality of life [1]. Thus, PPHC should begin at the time of diagnosis of a LLC and entail planning for the future. To ensure that the patients and/or parents’ wishes are respected, PPHC aims to facilitate advance care planning (ACP) discussions and informed decision-making regarding end-of-life care. ACP is deemed as a process including the opportunity to make and sometimes modify decisions in a timely fashion in a multi-disciplinary team including patients, whenever possible, their families as well as health care professionals [2–4]. One important result of an ACP discussion are “medical orders for life-sustaining treatment” (MOLST) [5].

In these, the patients/parents specify, whether they wish for the whole range of resuscitation and medical interventions should a life-threatening situation occur (Full Code) or whether they wish for comfort care only (Do Not Attempt Cardio-Pulmonary Resuscitation (DNACPR)). Alternatively, they might wish for some invasive life-sustaining interventions (such as suctioning, mask ventilation, intubation) but not for others (treatment limitations).

Since end-of-life discussions and decisions for children, adolescents and young adults (CAYA) are challenging, several barriers to the process have been identified in the literature such as the recognition of the life-limiting nature of an illness, gaining consensus of medical opinion, and physician reluctance or feeling ill prepared and inadequately trained to undertake ACP discussions [6–11]. On the other hand, recent studies indicate that parents are interested in ACP and that such discussions are best repeated on a regular basis [12]. According to the most recent guidance from the National Institute for Health and Care Excellence (NICE), MOLST should be developed and recorded at an appropriate time for the current and future care of each child or young person with a LLC [13]. However, only very few studies on ACP discussions and the resulting MOLST in CAYA are available, which primarily focus on the parents’ views and needs or are restricted to inpatient hospice care [12, 14, 15]. What is more, these studies do not report the circumstances of the patients’ deaths, so that no comparison (and equally no prediction) can be drawn between the parents’ wishes and what actually happened at the moment of death.

Particularly, data on MOLST in the home setting and comparing MOLST in children and their families according to the four “Together for short lives” (TfSL) classification groups (Category 1: Life-threatening conditions for which curative treatment may be feasible but can fail; Category 2: Conditions in which premature death is inevitable; Category 3: Progressive conditions without curative treatment options; Category 4: Irreversible but non-progressive conditions causing severe disability, leading to susceptibility to health complications and likelihood of premature death) [16] are completely lacking. This differentiation between the four groups - which were first defined by the “International Meeting for Palliative Care in Children” (IMPaCCT) group in 2008 [17] - is highly relevant, as one would expect, that the wishes and decision making are different between the groups due to the widely varying underlying conditions and disease trajectories.

To expand our knowledge of ACP and MOLST in PPHC and community care, we first aimed to determine the number of CAYA in PPHC with any type of MOLST, second to compare these data between the four TfSL groups and third to analyze, whether there was a relationship between the content of the MOLST and the place of death.

## RESULTS

### The clear majority of all parents decided on a written MOLST

Our PPCT cared for 198 CAYA (for diagnoses please refer to Supplementary Table 1) during the study period; 95 (48.0%) patients were female, mean age at referral was 8.7 years (range 0.0–25.0 years, standard deviation 7.2 years). Most CAYA (119; 60.1%) were from German descent. Mean duration of care was 355 days (range 1–2754; standard deviation 525 days); and mean number of home visits was 12.5 (range 1–80, standard deviation 13.2 visits). Less than half of all patients are still alive (92; 46.5%); 85 (80.2%) out of the 106 CAYA who died did so at home/in hospice. Patients in TfSL groups 1/3/4, who died during the course of PPHC, had a younger age at the start of PPHC compared to all patients of the respective groups at the start of PPHC. Details on demographic data and TfSL group distribution can be found in Table 1.

**Table 1:** Demographic data of children, adolescents and young adults (*n* = 198) cared for by the PPCT between January 1, 2013 and September 15, 2016

	All children	TfSL group 1	TfSL group 2	TfSL group 3	TfSL group 4
Number	198	65	13	49	71
Gender, male (%)	103 (52%)	39 (60%)	6 (46%)	22 (45%)	36 (51%)
Age at referral of all children, median (range in years)	8.4 (0.0–25.0)	11.2 (0.0–22.5)	8.4 (0.2–23.9)	1.8 (0.0–24.2)	7.1 (0.1–25.0)
Age at referral of later diseased children, median (range in years)	6.8 (0.0–25.0)	10.0 (0.0–22.5)	11.6 (0.2–23.9)	1.2 (0.1–24.2)	2.1 (0.1–25.0)
Duration of palliative care, median (range in days)	122 (1–2754)	39 (1–441)	91 (3–545)	288 (1–2248)	268 (2–2754)
Home visits, median (range)	8 (1–80)	7 (1–41)	6 (1–20)	10 (1–80)	8 (1–64)
No. of deceased children (%)	106 (54%)	56 (86%)	7 (54%)	21 (43%)	22 (31%)
Place of death, *n* (%)					
At home	67 (63%)	45 (80%)	5 (71%)	8 (38%)	9 (41%)
In hospice/PCU	16 (15%)	6 (11%)	1 (14%)	3 (14%)	6 (27%)
In hospital	23 (22%)	5 (9%)	1 (14%)	10 (48%)	7 (32%)
Age at death, median (range in years)	7.1 (0.1–27.6)	10.4 (0.1–22.6)	11.2 (0.2–24.1)	1.9 (0.1–26.7)	3.0 (0.2–27.6)

Nineteen (9.6%) out of 198 families in total did not wish to set down a written MOLST, 3 patients each of TfSL groups 2 and 3, and 10 patients of TfSL group 4. Of those 19 patients, 7 died, of whom two at home and five at hospital. In 10 patients care was interrupted for various reasons, two patients are still in PPHC at the end of this study.

In all those cases, when the adolescents were mentally able to, they were part of the ACP discussions and their wishes were followed. Nineteen patients were over the age of 18 years at the start of PPHC. Of those, nine had a legal guardian (in most cases the parents), who signed the MOLST, in eight cases it was the young adults themselves (for two patients this information is lacking). During PPHC, another 12 patients turned eighteen, of those, eleven had a legal guardian and one patient was able to decide for himself.

Looking at the different age groups (across all TfSL groups) of all patients in PPHC, parents of young children (i.e. less than 12 months of age) less often decided on a DNACPR at the start of PPC (8/43, 18.6%) compared to parents of children older than 10 years of age (40/89, 44.9%). However, when not considering TfSL group 1 patients (as most children with a terminal oncological disease are older than 1 year) numbers were not different between the age groups (less than one year 6/40 [15.0%] vs. older than ten years 6/55 [10.9%]).

The following analyses were performed with TfSL group 2–4 patients only. Regarding ethnicity, there was a trend for parents of German origin to decide on a DNACPR (13/79, 16.5%) slightly more often compared to non-German parents (5/54, 9.3%).

When looking at those patients, who were in PPHC less than 177 days (less than half the mean duration of care, which was 355 days), 13/57 (22.8%) initially decided on a DNACPR. Of all patients, who were in PPHC less than 355 days, 15/76 (19.7%) decided on a DNACPR. Conversely, only 3/57 (5.3%), who were in care for longer than 355 days wished for a DNACPR and even only 1/33 (3.0%), who were in care for longer than 710 days (twice the mean of 355 days).

### MOLST significantly differed between the four TfSL groups

**Figure 1:**
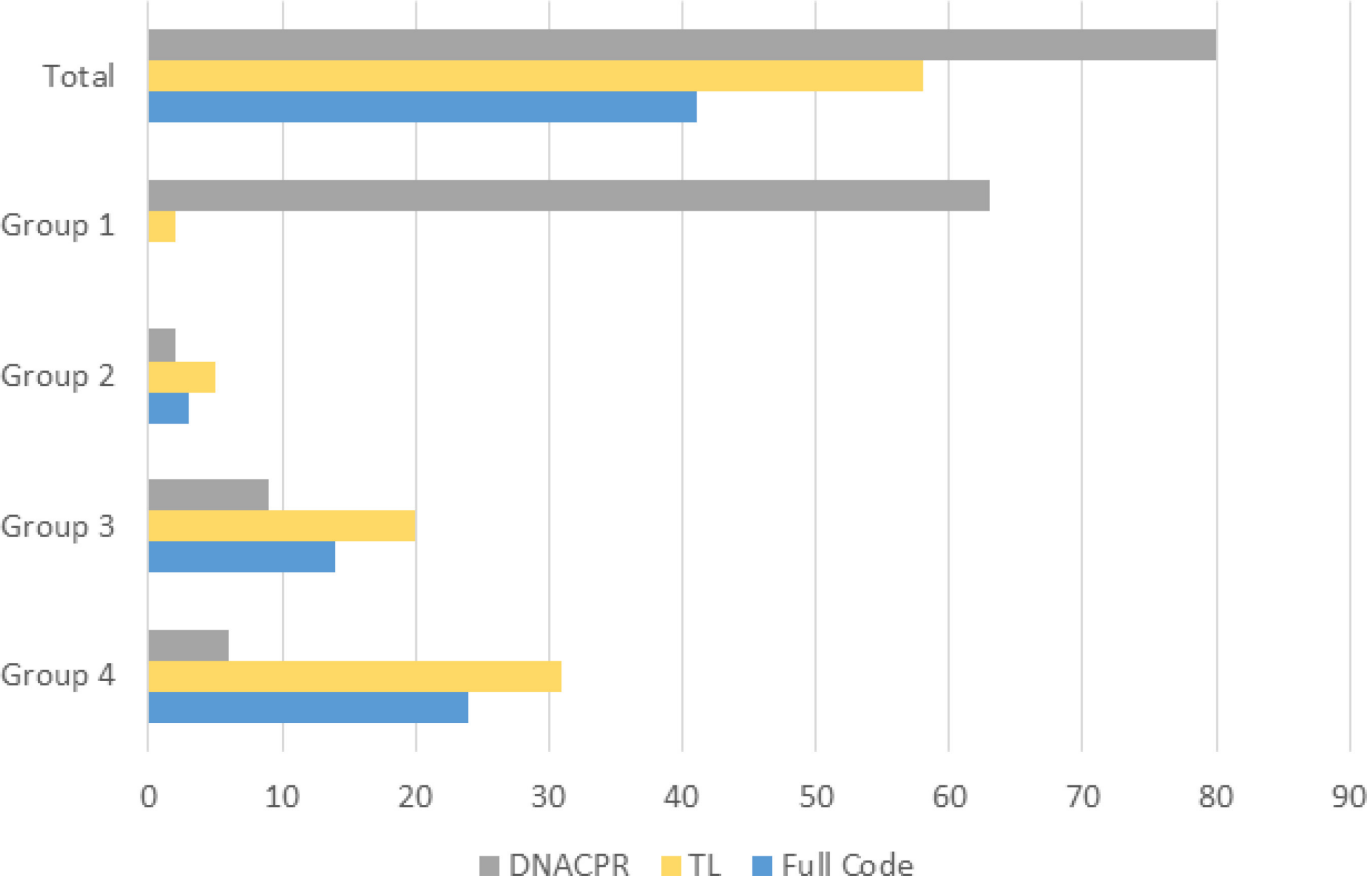
Overview on MOLST in 179 children, adolescents and young adults and broken down according to the TfSL groups

Thus, data on MOLST were available in 179 (90.4%) patients; 80 (44.7%) had a DNACPR specifying comfort measures only, 58 (32.4%) had TL, and 41 (22.9%) patients were considered as Full Code (Figure 1).

**Figure 2:**
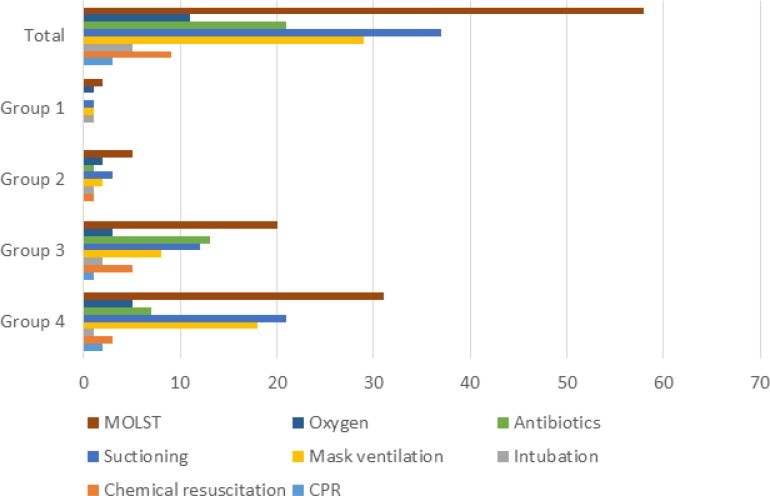
Details on MOLST broken down according to the TfSL groups

Looking into the MOLST in more detail of those 58 patients/families, who opted for TL, the minority of the families decided for CPR (3; 5.2%), chemical resuscitation (9; 15.5%), and intubation/ventilation (5; 8.6%). Much more frequent were the wish for medical interventions such as the use of antibiotics (20; 34.5%), (bag) mask ventilation (28; 48.3%), use of oxygen (10; 17.2%), and use of suction equipment (36; 62.1%) (Figure 2).

Most parents decided fast on the content of the MOLST, median time to a decision was 0 days, with 99 (55.3%) parents deciding at the time of starting PPHC and 150 (83.8%) having decided within the first 100 days. Time to deciding on a DNACPR was (except for TfSL group 2) shorter in all groups than time to deciding on TL or a Full Code. If parents took longer than 100 days to decide on the MOLST, they were more likely to choose Full Code (17/29 (58.6%)).

In 63 (35.2%) patients (exclusively in patients with cancer), a DNACPR was determined at acceptance into PPHC. For all other patients (116; 64.8%), median time from acceptance for PPHC to the implementation of MOLST was 27 days (range 0–2351) and 88 days (range 0–2351) for a DNACPR. Looking into those 29 cases (11x group 3, 18× group 4) in which a MOLST was determined later than 100 days, revealed that 17 families (58.6%) opted for a Full Code (45.5% group 3, 66.7% group 4). The percentage of families wanting Full Code rose to 64.7% when considering only those MOLST, who were first formulated more than 200 days into the start of care (17 cases). The median number of ACP discussions in non-oncologic patients was 2 (range 1–9). General information on MOLST are given in Table 2. In summary, TfSL group 1 patients wished for a DNACPR more often compared to groups 2–4 (*p* value < 0.001). Similarly, groups 2–4 chose Full Code more often than group 1 (*p* value < 0.001).

**Table 2:** General information on written medical orders for life sustaining measures (MOLST) in 179 children, adolescents and young adults and broken down according to the TfSL groups

	Total	TfSL group 1	TfSL group 2	TfSL group 3	TfSL group 4
Written MOLST available based on all children	179 (90.4%)	65 (100%)	10 (76.9%)	43 (87.8%)	61 (85.9%)
Time from acceptance into PPC to MOLST, median (range in days)	0 (0–2351)	14.5 (0–29)	8.5 (0–63)	56 (0–545)	25 (0–2351)
Time from acceptance into PPC to DNACPR, median (range in days)	0 (0–144)	0 0	17 (0–37)	0 (0–105)	0 (0–144)
No. of ACP discussions, median (range)	1 (1–7)	1	1 (1–4)	2 (1–9)	2 (1–7)
Written MOLST rescinded, no.	14	0	2	5	7
No. of children who died at home/in hospice with	83	53	4	11	15
DNACPR	63	53	2	4	4
Treatment limitations	20	0	2	7	11
Full Code	0	0	0	0	0
No. of children who died in hospital with	16	3	1	7	5
DNACPR	6	3	0	2	1
Treatment limitations	5	0	1	2	2
Full Code	5	0	0	3	2

### The MOLST mostly remains constant over time

Fourteen (7.8%) families changed the MOLST during PPHC. Instead of a Full Code, four families changed to TL and one family changed to a DNACPR. An additional three families had initially opted for TL; two of those changed to a DNACPR and one did not wish for chemical resuscitation anymore. In all cases, this decision was based on a (slowly) deteriorating condition of the patient, in some cases also on multiple hospital admissions, which the parents did not wish for their children anymore. Five families opted for more intensive treatment; one initially opted for more intensive treatment but later reduced treatment intensity again. The PPHC of two children, in whom the parents opted for more intensive treatment, was later paused due to a notable clinical stabilization.

### The content of the MOLST predicts the actual place of death

Median duration of care of those 99 (55.3%) CAYA who died during the study period was 50 days (range 1–1574). Of these 99 deaths, 82 (83.8%) occurred at home/in hospice with 63 patients having a DNACPR which was followed in 62 (98.4%) cases. Only one family during the entire study period contacted emergency services for resuscitation: however, the resuscitation was unsuccessful. Twenty-two (67.7%) patients with TL died at home/in hospice without invasive therapies at the end of life. Sixty-three with a DNACPR (91.3%) died at home. As opposed to this, none of the five Full Code patients died at home, four of them even died in an intensive care unit (ICU) setting.

**Figure 3:**
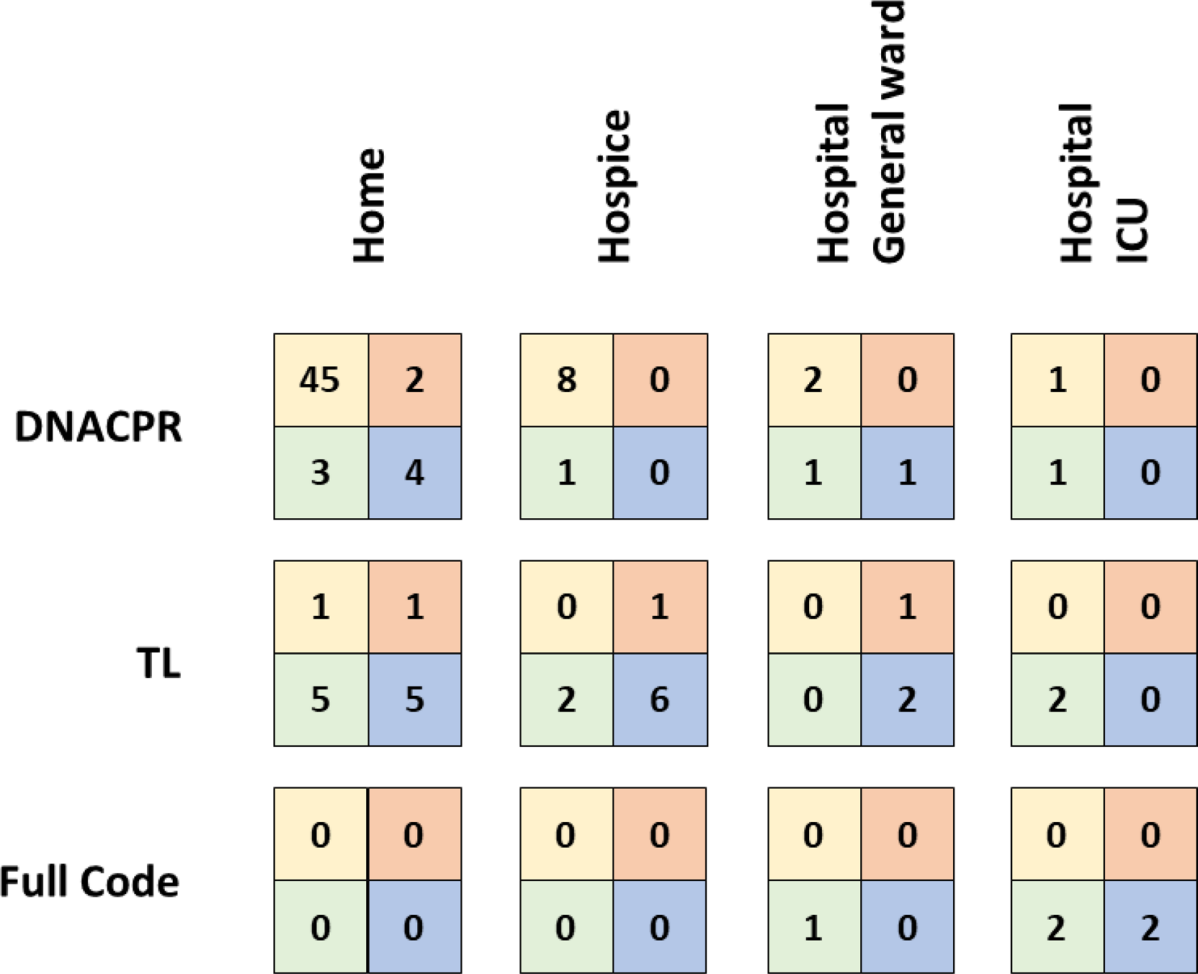
Overview on MOLST and outcome in 99 children and young adults, who died during the study period The boxes represent the four TfSL groups, left upper square group 1, right upper square group 2, left lower square group 3, right lower square group 4.

Out of the 16 patients, who died in hospital, 11 had a DNACPR or TL. Reasons for readmission included respiratory problems (mostly pneumonias) but also intractable vomiting and status epilepticus. Although all patients received some interventions (including antibiotics and oxygen via face mask), only three patients were intubated and only in one patient resuscitation was attempted. An overview on MOLST and outcome in 99 children and young adults, who died during the study period, is given in Figure 3.

## DISCUSSION

The clear majority of families (90.4%) in PPHC decided on MOLST. Only few families (7.8%) subsequently changed the MOLST in a relevant manner (i.e. concerning chemical resuscitation and/or intubation and/or CPR). Whereas most CAYA with a DNACPR died at home, all Full Code patients died in hospital. Significantly more TfSL group 1 families decided on a DNACPR, whereas most Full Codes were present in groups 3 and 4.

Advance care planning seeks to avoid unnecessary suffering and to ensure care conforming to the patients and their families’ preferences based on clear and timely information [15, 18–21]. Our study indeed shows that most families in PPHC opt for MOLST. This is in contrast to a previous report of the Canuck Place Children’s Hospice program, in which only half of the families in hospice decided on MOLST [14]. Noteworthy, MOLST at acceptance into PPHC was neither required by the Canuck nor by our team. However, at referral of oncological patients to PPHC, which contributed to about one third of our patients, the extremely dismal prognosis and the lack of any medical indication for resuscitation was clearly stated by the members of our PPCT. Thus, almost all parents followed the explicit recommendation to a DNACPR. This process substantially differed from practice policy in children with other life-limiting conditions (TfSL groups 2–4). The unpredictable disease trajectory and, thus, prognosis caused uncertainty about medically indicated interventions even in the members of the PPCT. Thus, ACP discussions were on the one hand performed with a more open outcome and on the other hand substantially less restrictive treatment recommendations were made to the parents. Lotz et al. previously reported on professionals’ discomfort and uncertainty regarding end-of-life decisions and MOLST [6]. However, these less restrictive treatment recommendations in groups 2–4 might also be discussed critically, as parents might feel, that if they decided on a DNACPR, they had to carry the burden of this decision alone.

Frequent emergency department visits increase parents’ interest in creating a written care plan [22] and regular conversations about MOLST with a constant contact person are important to the parents [4, 6, 12, 23, 24]. To avoid rushed decisions based on insufficient information and respite, ACP discussions should be integrated in a timely fashion. However, in critically ill children and young adults who were referred to PPHC usually from ICU [24, 25], ACP discussions were started just at that moment. Otherwise, these discussions were postponed to at least the second home visit to initially get to know each other before. This practice led to an average time to MOLST in non-oncological patients of 29 days, which is equal to that reported by Siden et al. [14]. Although this may seem a short period, no parents were rushed into advance care discussions and these were always postponed, if the parents asked for it (regardless the reason). Moreover, in our study, most patients of TfSL group 2–4 were enrolled in PPHC in clinically critical situations or phases of acute deterioration and, thus, most parents were already confronted with the issue of life-sustaining treatment.

In most of the CAYA in whom MOLST specified TL, these interventions were anyway common practice in daily care due to pre-existing conditions, and, thus, decided to be continued in mutual agreement between both the parents and healthcare professionals [26].

In most of the families with a DNACPR the patients died at home/in hospice without unwished interventions and all deceased patients with a Full Code died in hospital. Siden et al. previously reported, that for in-hospice deaths MOLST were followed in almost all cases [14].

Especially in CAYA with life-limiting conditions with a long(er) or unpredictable disease trajectory, treatment goals might change over time. This might depend on factors such as severity of acute deterioration as well as incomplete recovery and enable parents to shift treatment goals and, thus, decisions on MOLST [26, 27]. And indeed, 8% of all families changed MOLST during PPHC, mostly limiting interventions. However, despite the fact, that the MOLST were rarely changed, regular discussions on ACPs should continue as they constitute an important part of PPHC. Additionally, having their child die at home is not an option for some families and, thus, dying in hospital might represent the desired option in some families.

To the best of our knowledge, this is the first study reporting on outcome of MOLST in CAYA with a focus on the home setting and differences between TfSL groups. It demonstrates that at least in our cohort, the end-of-life phase of CAYA runs in accordance with the previously determined wishes of most families. However, due to the retrospective nature of our study, we were unable to capture the parents’ and healthcare professionals’ perspectives and whether end-of-life care ultimately conformed to the families’ perceptions [23, 28, 29]. Furthermore, we acknowledge that the single center analysis limited the ability to fully explore some hypotheses, e.g. the impact of the PPCT on parents’ decisions at the end of life [30, 31]. Since our PPCT represents one of the leading teams in Germany regarding the implementation of MOLST our results may draw an overoptimistic picture of the current ACP practice.

ACP discussions and MOLST in children is a difficult task both for healthcare providers as well as parents but is not intended to take away hope [32]. To ensure this, it should be integrated in discussions on treatment and care goals as a regular aspect of PPHC in a timely manner. Parents not only regard these discussions as an important aspect of their children’s care but – as our data surmise – also are able to set down a MOLST, which they then follow at the end of life. However, as the families’ decisions on MOLST are significantly different between the four TfSL groups [23, 24], healthcare professionals need to consider the different and sometimes unpredictable disease trajectories of the various life-limiting conditions when performing ACP discussions.

Regular ACP discussions appear to have a significant impact on outcome at least in our setting. Future research should focus on how parents felt about their decisions and the actual circumstances of the deaths to increase the benefit for the families.

## MATERIALS AND METHODS

The study cohort of our retrospective study were all CAYAs (0–25 years), who were cared for by our specialized pediatric palliative care team (PPCT) based at the Children’s University Hospital. Our PPCT was founded more than 30 years ago, initially members of the team acted on a voluntary basis. The team grew over the years and now consists of several pediatricians with a specialization in palliative care, nurses with a specialization in palliative care nursing, and a social worker, among others. The PPCT serves a very large region and represents one of the largest teams in Germany. The study period was from January 1st 2013 to September 15th 2016. The time between the start of PPHC and the date of data collection, interruption of care or death, as applicable, was analyzed as duration of PPHC. Care was interrupted in cases, in which either the child’s condition was stable for a long period of time and/or the health insurance denied further coverage of costs of PPHC.

MOLST were recorded in three categories, Full Code (when the parents wished for the whole range of resuscitation and medical interventions), treatment limitations (TL; a combination of any of the following [but with at least one exception, as otherwise it would have been Full Code]: oxygen, antibiotics (orally/intravenously), suctioning, mask ventilation, intubation, chemical resuscitation [defined as selectively using medication only and not intubation, defibrillation or cardio compression]), and Do Not Attempt Cardio-Pulmonary Resuscitation (DNACPR).

When looking at potential changes in MOLST, we classified all changes as major, if chemical resuscitation and/or intubation and/or CPR were altered. Reasons for changing MOLST were documented in the charts.

We applied a one-sided exact fisher-test (R Version 3.3.0) to compare group 1 versus the sum over the groups 2–4 (i.e. not-group 1).

## SUPPLEMENTARY MATERIALS TABLE





## Abbreviations

ACP: advance care planning
CAYA: children: adolescents and young adults
DNACPR: Do Not Attempt Cardio-Pulmonary Resuscitation
LLC: life-limiting condition
MOLST: medical orders for life-sustaining treatment
PPCT: pediatric palliative care team
PPHC: pediatric palliative home care
TfSL: Together for Short Lives
TL: treatment limitations
