# Supplementary material for: Advance care planning and outcome in pediatric palliative home care
Source: Oncotarget. 2018 Apr 3;9(25):17867–75. doi: 10.18632/oncotarget.24929 (PMC5915160; doi:10.18632/oncotarget.24929)
Supplement: Supplementary file 1 [file oncotarget-09-17867-s001.pdf]

Advance care planning and outcome in pediatric palliative home care

SUPPLEMENTARY MATERIALS

Supplementary Table 1: Overview on the patients' diagnoses in the four TfSL groups. See Supplementary_Table_1
