# Supplementary material for: Advance care planning and outcome in pediatric palliative home care
Source: Oncotarget. 2018 Apr 3;9(25):17867–75. doi: 10.18632/oncotarget.24929 (PMC5915160; doi:10.18632/oncotarget.24929)
Supplement: Supplementary file 2 [file oncotarget-09-17867-s002.docx]

**Supplementary Table 1: Overview on the patients’ diagnoses in the four TfSL groups**

| **TfSL group** | **Diagnoses** | **Count** |
| --- | --- | --- |
| 1 | Acute lymphoblastic leukemia | 3 |
| 1 | Acute myeloid leukemia | 2 |
| 1 | Astrocytoma | 5 |
| 1 | Atypical teratoid/ rhabdoid tumor | 3 |
| 1 | Chordoma | 1 |
| 1 | Congenital brain tumor | 1 |
| 1 | Desmoplastic small round cell tumor | 1 |
| 1 | Diffuse intrinsic pontine glioma | 6 |
| 1 | Ependymoblastoma | 1 |
| 1 | Ependymoma | 1 |
| 1 | Ewing sarcoma | 4 |
| 1 | Ganglioglioma | 1 |
| 1 | Glioblastoma | 8 |
| 1 | Glioma | 1 |
| 1 | Intracranial germ cell tumor | 2 |
| 1 | Medulloblastoma | 2 |
| 1 | Nephroblastoma | 1 |
| 1 | Neuroblastoma | 6 |
| 1 | Osteosarcoma | 5 |
| 1 | Pleuropulmonary blastoma | 1 |
| 1 | Primitive neuroectodermal tumor | 1 |
| 1 | Renal cell carcinoma | 1 |
| 1 | Rhabdoid tumor | 1 |
| 1 | Rhabdomyosarcoma | 5 |
| 1 | Squamous cell carcinoma | 1 |
| 1 | T-cell lymphoma | 1 |
| 2 | Biliary atresia | 2 |
| 2 | Double outlet right ventricle | 1 |
| 2 | Duchenne muscular dystrophy | 3 |
| 2 | Hypoplastic left heart syndrome | 2 |
| 2 | Klippel-Trénaunay-Weber syndrome | 1 |
| 2 | Louis Bar syndrome | 1 |
| 2 | Marfan syndrome | 1 |
| 2 | Unknown syndrome with multiple malformations (mainly cardiac) | 2 |
| 3 | 3-methylglutaconic aciduria | 1 |
| 3 | Alexander disease | 2 |
| 3 | ARC syndrome | 1 |
| 3 | Canavan disease | 1 |
| 3 | Congenital myopathy | 1 |
| 3 | Crabbe disease | 1 |
| 3 | EARS2 mutation | 1 |
| 3 | Evans syndrome | 1 |
| 3 | GM2 gangliosidosis | 1 |
| 3 | Immunodeficiency | 1 |
| 3 | Kohlschütter syndrome | 1 |
| 3 | Leigh's disease | 2 |
| 3 | Medium-chain acyl-CoA dehydrogenase (MCAD) deficiency | 1 |
| 3 | Metachromatic leukodystrophy | 3 |
| 3 | Mitochondrial disease of unknown etiology | 4 |
| 3 | Molybdenum cofactor deficiency | 1 |
| 3 | Mucolipidosis type II | 2 |
| 3 | Nemaline myopathy | 1 |
| 3 | Neurodegenerative disease of unknown etiology | 1 |
| 3 | Neuronal ceroid lipofuscinosis | 2 |
| 3 | Nonketotic hyperglycinemia | 2 |
| 3 | Osteopetrosis | 1 |
| 3 | Pearson syndrome | 2 |
| 3 | Rett syndrome | 1 |
| 3 | Sphingolipidosis | 2 |
| 3 | Spinal muscular atrophy | 8 |
| 3 | Sulfite oxidase deficiency | 1 |
| 3 | TMEM70 mutation | 1 |
| 3 | Unclear syndrome (mainly metabolic) | 2 |
| 4 | 18q syndrome | 1 |
| 4 | Acute disseminated encephalomyelitis | 1 |
| 4 | Aicardi Goutieres syndrome | 1 |
| 4 | Arnold Chiari malformation | 1 |
| 4 | Bannayan-Riley-Ruvalcaba syndrome | 1 |
| 4 | Battered child syndrome | 2 |
| 4 | Campomelic dysplasia | 2 |
| 4 | Cerebral palsy of unknown etiology | 4 |
| 4 | Cornelia-de-Lange syndrome | 1 |
| 4 | Encephalitis of unknown etiology | 2 |
| 4 | Enterococcal meningitis | 1 |
| 4 | GABRB3 mutation | 1 |
| 4 | Herpes simplex encephalitis | 2 |
| 4 | Hydrocephalus-Agyria-Retinal dysplasia | 1 |
| 4 | Hypoxic brain injury due to near drowning | 6 |
| 4 | Intraventricular hemorrhage | 2 |
| 4 | Left cerebral agenesis | 1 |
| 4 | Lissencephaly | 1 |
| 4 | Miller Dieker syndrome | 1 |
| 4 | Mycoplasma pneumoniae encephalitis | 1 |
| 4 | Neonatal sepsis | 2 |
| 4 | Noonan syndrome | 1 |
| 4 | Ohtahara syndrome | 1 |
| 4 | Oral-facial-digital syndrome | 1 |
| 4 | Perinatal asphyxia | 8 |
| 4 | Pontocerebellar hypoplasia | 1 |
| 4 | Posttraumatic brain injury | 3 |
| 4 | Suspected battered child syndrome | 1 |
| 4 | Trisomy 18 | 5 |
| 4 | Trisomy 9 | 1 |
| 4 | Unclear syndrome with multiple malformations | 14 |
